# Microporosity and CO_2_ Capture Properties of Amorphous Silicon Oxynitride Derived from Novel Polyalkoxysilsesquiazanes

**DOI:** 10.3390/ma11030422

**Published:** 2018-03-13

**Authors:** Yoshiaki Iwase, Yoji Horie, Sawao Honda, Yusuke Daiko, Yuji Iwamoto

**Affiliations:** 1 Applied Research Laboratory, General Center of Research and Development, Toagosei Co., Ltd., 8, Showa-cho, Minato-ku, Nagoya 455-0026, Japan; yoshiaki_iwase@mail.toagosei.co.jp (Y.I.); youji_horie@mail.toagosei.co.jp (Y.H.); 2 Department of Life Science and Applied Chemistry, Graduate School of Engineering, Nagoya Institute of Technology, Gokiso-cho, Showa-ku, Nagoya 466-8555, Japan; honda@nitech.ac.jp (S.H.); daiko.yusuke@nitech.ac.jp (Y.D.)

**Keywords:** silicon oxynitride, amorphous state, microporosity, CO_2_ adsorption capacity, polymer-derived ceramics (PDCs)

## Abstract

Polyalkoxysilsesquiazanes ([ROSi(NH)_1.5_]_n_, ROSZ, R = Et, nPr, iPr, nBu, sBu, nHex, sHex, cHex, decahydronaphthyl (DHNp)) were synthesized by ammonolysis at −78 °C of alkoxytrichlorosilane (ROSiCl_3_), which was isolated by distillation as a reaction product of SiCl_4_ and ROH. The simultaneous thermogravimetric and mass spectrometry analyses of the ROSZs under helium revealed a common decomposition reaction, the cleavage of the oxygen–carbon bond of the RO group to evolve alkene as a main gaseous species formed in-situ, leading to the formation of microporous amorphous Si–O–N at 550 °C to 800 °C. The microporosity in terms of the peak of the pore size distribution curve located within the micropore size range (<2 nm) and the total micropore volume, as well as the specific surface area (SSA) of the Si–O–N, increased consistently with the molecular size estimated for the alkene formed in-situ during the pyrolysis. The CO_2_ capture capacity at 0 °C of the Si–O–N material increased consistently with its SSA, and an excellent CO_2_ capture capacity of 3.9 mmol·g^−1^ at 0 °C and CO_2_ 1 atm was achieved for the Si–O–N derived from DHNpOSZ having an SSA of 750 m^2^·g^−1^. The CO_2_ capture properties were further discussed based on their temperature dependency, and a surface functional group of the Si–O–N formed in-situ during the polymer/ceramics thermal conversion.

## 1. Introduction

Micro and mesoporous structure formation through the polymer-derived ceramics (PDCs) [1,2] route has received increasing attention as an attractive ceramic processing route to develop gas separation membranes, gas sorbents and catalysts with thermally and/or chemically stable amorphous systems such as silicon nitride [3], silicon carbide [4,5,6,7,8,9], silicon carbonitride (Si–C–N) [10], silicon oxycarbide (Si–O–C) [11,12,13], silicon oxycarbonitride (Si–O–C–N) [14,15,16] and other quaternary Si–M–C–N (M=B, [17,18], Ni [19]). During the crosslinking and subsequent high-temperature pyrolysis of polymer precursors, by-product gases such as CO_2_, CH_4_, NH_3_ and H_2_ were detected, and the microporosity in the amorphous PDCs could be assigned to the release of the small gaseous species formed in-situ [14,15,16,20,21,22,23]. 

Recently, we reported a novel single source precursor for a silicon oxynitride (Si–O–N) system, ethoxysilsesquiazane ([EtOSi(NH)_1.5_]_n_, EtOSZ) [24]. Under inert atmosphere up to 800 °C, this polymer exhibited a unique thermal decomposition behavior: a cleavage of the oxygen–carbon bond of the EtO group to evolve ethylene as a main gaseous species formed in-situ, which led to the formation of amorphous Si–O–C–N with an extremely low carbon content (1.1 wt %) compared to the theoretical EtOSZ (25.1 wt %). Thus, the EtO group can be expected to act as a “sacrificial template” to afford a micro and mesoporous material as previously reported for the organo-substituted polysilazanes [22,23,25]. 

In this study, various alkoxy group-functionalized silsesquiazane ([ROSi(NH)_1.5_]_n_, ROSZ) analogues were synthesized. The polymer/ceramics thermal conversion under a nitrogen flow was performed on the polymers. The thermal conversion behaviors were analyzed in-situ by simultaneous thermogravimetry-mass spectrometry analysis. The relationships between the alkoxy group, gaseous species formed in-situ during the thermal conversion and the microporosity in the resulting amorphous Si–O–N materials are discussed. 

Moreover, as our initial feasibility study on their application, CO_2_ capture properties of the as-synthesized amorphous Si–O–N materials were examined. Compared with the conventional CO_2_ capture process using amine scrubbing, the novel solid sorbent-based process offers several potential advantages such as low equipment corrosivity, less toxicity, and cost-effective regenerability of the sorbent that can be expected to significantly lower the energy input during regeneration and overcome the issues associated with the use of traditional aqueous amine sorbents [26,27]. 

CO_2_ sorption can be explained by physical and/or chemical adsorption on solid sorbents. Physical adsorption (physisorption) involves weak van der Waals forces and decreases with increasing temperature, whereas chemical adsorption (chemisorption) implies the formation of chemical bonding between the sorbent and CO_2_, thus being more stable to temperature. On the other hand, current basic technologies for the regeneration of the sorbents are pressure swing adsorption (PSA), vacuum swing adsorption (VSA) and temperature swing adsorption (TSA) [28,29]. In a VSA process, CO_2_ is adsorbed at close to ambient pressure and released under reduced pressure. Thus, VSA is suited for post-combustion streams, for instance CO_2_ capture from flue gas produced by coal-fired power plants, which are recognized as the largest source of CO_2_ emission in the world. The CO_2_ concentration in the flue gas is less than 15% at 1 bar and above 20 °C. Accordingly, for the VSA systems, the solid sorbents with a chemisorption mechanism can be expected to show high CO_2_ capture capacity due to the fact that they can adsorb CO_2_ selectively in the presence of other gases and can operate at high temperatures. However, these advantages of the chemisorption mechanism are counteracted by the high-energy consuming regeneration at high temperatures. To overcome this drawback, recently, novel amine-impregnated solid solvents were designed and synthesized based on the density functional theory calculation, and their low-temperature regeneration was demonstrated under saturated water vapor pressure (60 °C, 20 kPa). However, this system was specifically effective at a site where waste steam was available [27]. Thus, most of the studies on VSA systems are solid sorbents with physisorption mechanisms such as zeolites [30,31], activated carbons [32,33,34] and metal-organic frameworks [26,35], having large CO_2_ capture capacities (3.8–8.6 mmol·g^−1^ at 0 to 20 °C) in the low pressure region up to 1 bar, and currently they are under development. 

In the case of PSA, the adsorption process is operated at high pressures, typically ranging from 8 to 28 bar [36,37], and the PSA systems for pre-combustion CO_2_ capture are especially attractive for solid sorbents to adsorb CO_2_ efficiently since the partial pressure of CO_2_ in the pre-combustion streams such as steam-methane reforming (SMR) off-gas is high, in the order of several atmospheres [38,39]. For this system, zeolite 13X and NaY with large CO_2_ capture capacity at moderate temperatures have been used, but regeneration of the zeolites requires very low vacuum and/or high-temperature. Thus, the majority of research on developing novel solid sorbents has focused on the cost-effective regenerability as well as high CO_2_ capture capacity. In this regard, activated carbons [40] and one of the metal-organic frameworks, UTSA-16 [41], have been suggested as candidate solid sorbents for the PSA systems [39].

Recently, microporous structure controlling in nitride-based amorphous solid sorbents has been proposed as an alternative approach to harmonize CO_2_ capture capacity and regenerability. Zhao et al. reported synthesis of porous carbon nitride (CN) spheres for CO_2_ capture [42]. The resulting CN spheres possessed—at 1 bar—a CO_2_ capture capacity of 2.90 at 25 °C and 0.97 mmol·g^−1^ at 75 °C, superior to those of the pure carbon materials with an analogous porous structure. In addition to the abundant nitrogen-containing basic groups and the hierarchical mesostructure with a relatively high Brunauer–Emmett–Teller specific surface area, it was suggested that a large number of micropores and small mesopores could contribute to the enhanced CO_2_ capture capacity, owing to the capillary condensation effect. Schitco et al. also reported that polymer-derived ultra-microporous amorphous silicon nitride showed an excellent CO_2_ capture capacity of 2.35 mmol·g^−1^ at 0 °C and 1 bar. They proposed that the observed high CO_2_ storage capacities could be achieved in materials with high amount of ultra-micropore volume [15].

In this study, CO_2_ capture capacities at 0 to 40 °C of the present ROSZ-derived microporous amorphous Si–O–N materials were evaluated and briefly compared with those in literature, then the capture behaviors under CO_2_ atmosphere were in-situ analyzed by using thermogravimetry and infrared spectroscopy.

## 2. Experimental Section

### 2.1. Precursor Synthesis

**Figure 1:**
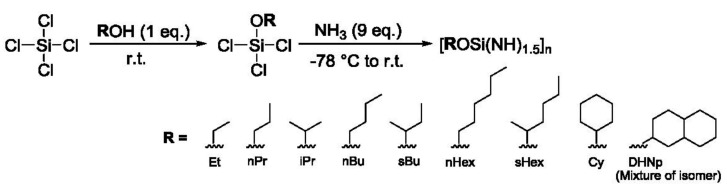
Synthetic route of ROSZs and the chemical structure of each alkyl in alcohols.

The handling of all the reagents and products in this study was performed under an inert atmosphere of pure nitrogen (N_2_). The starting alcohols, tetrachlorosilane and reaction solvent of dry tetrahydrofuran (reagent grade) were purchased from Wako Pure Chemicals Industry, Osaka, Japan, and used without further purification. The purity of gaseous ammonia used in this study was >99.9% (Sumitomo Seika Chemicals, Osaka, Japan). Eight kinds of [ROSi(NH)_1.5_]_n_ analogues were synthesized by varying alcohol species through the common synthetic route (Figure 1) in the same manner and under the same conditions as previously reported for the synthesis of EtOSZ [24]. After the first reaction, the ROSiCl_3_ was isolated by distillation. The purity of the distillated ROSiCl_3_ was monitored by gas chromatography (GC) analysis, and the ROSiCl_3_ fraction with the purity higher than 95% was collected. The recovery rate of each ROSiCl_3_ was evaluated as the amount of the collected fraction relative to the total fraction. The yield after ammonolysis at second step was determined by measuring the weight of the reaction product, then the total yield of each ROSZ analogue was calculated from those values listed in Table 1.

### 2.2. Pyrolysis and Heat Treatment

The synthesized ROSZ was placed on an alumina tray and pyrolyzed in a quartz tube furnace under flowing N_2_ (200 mL/min.) by heating from room temperature to 550–800 °C with a heating rate of 5 °C/min, maintaining the maximum temperature for an additional 1 h and finally furnace cooling down to room temperature to give a product as a solid. The ceramic yield and the appearance of the 800 °C-pyrolyzed samples were listed in Table 2.

### 2.3. Characterizations

^13^C and ^29^Si solid state nuclear magnetic resonance (NMR) spectra for the as-synthesized and the pyrolyzed-ROSZ samples were acquired using magic angle spinning (MAS) with a rotation frequency of 15 kHz (Model ECA-400, JEOL, Tokyo, Japan) at room temperature. The resonance frequencies for the ^13^C– and ^29^Si–NMR spectra recorded in this study were 100 and 79.5 MHz, respectively. The chemical shifts of the peak signals in the ^13^C– and ^29^Si–NMR spectra were quoted relative to the signals of adamantine (29.5 pm) and 3-(trimethylsilyl) propionic acid sodium salt (2 ppm), respectively. 

The attenuated total reflection–infra red (ATR-IR) spectra were recorded on the as-synthesized and the pyrolyzed ROSZ with a diamond prism under an incidence angle of 45° (Model Spectrum 100, Perkin Elmer, Waltham, MA, USA). 

The thermal behaviors up to 1000 °C were studied by thermogravimetric (TG) analysis in N_2_ with a heating rate of 10 °C/min (Model TG-DTA 6300, Hitachi High Technologies Ltd., Tokyo, Japan), and simultaneous TG-mass spectrometry (MS) analyses (Model STA7200, Hitachi High Technologies Ltd., Tokyo, Japan/Model JMS-Q1500 GC, JEOL, Tokyo, Japan). The measurements were performed under flowing helium (100 mL/min.) with a heating rate of 10 °C/min.

To examine the effect of the molecular size of alkenes formed in-situ on the porosity formation, molecular structure calculations were performed by Spartan’14 using the basis set of ωB97-XD/6-31G*.

Elemental analyses were performed on the pyrolyzed samples for oxygen, nitrogen, and hydrogen (inert-gas fusion method, Model EMGA-930, HORIBA, Ltd., Kyoto, Japan), and carbon (non-dispersive infrared method, Model CS844, LECO Co., St. Joseph, MI, USA). The silicon content in the samples was calculated as the difference of the sum of the measured C, N, O and H content to 100 wt %.

X-ray diffraction (XRD) measurements were performed on the pyrolyzed samples (Model X’pert Pro α1, Philips Ltd., Amsterdam, The Netherlands). 

Textural properties of the pyrolyzed samples were measured by gas adsorption/desorption isotherms (Model Belsorp Max, BEL Japan Inc., Osaka, Japan) using argon (Ar) as a probe molecule with relative pressures ranging from 0 to 0.99. Specific surface area (SSA) was calculated from the isotherm data using the Brunauer-Emmett-Teller (BET) method. The micropores (R_pore_ < 2.0 nm) were characterized by the Saito-Foley (SF) method [43]. 

To study the CO_2_ affinity of the ROSZ-derived amorphous Si–O–N, TG analysis under CO_2_ atmosphere was conducted (Model TG8120, Rigaku, Tokyo, Japan). Dry CO_2_ was used for the runs and high purity Ar was used as the purging gas for sorbent regeneration. CO_2_ adsorption-desorption measurements were conducted at 40 °C under continuous run Ar (40 min), CO_2_ (40 min) then Ar (40 min), respectively, according to a published procedure [44,45]. CO_2_ adsorption-desorption isotherms were also carried out at 0, 20 and 40 °C (Model Belsorp Max, BEL Japan Inc., Osaka, Japan). All samples were dried 1 h at 120 °C under vacuum to remove moisture and CO_2_ adsorbed from air prior to the above characterization experiments. For each measurement, the adsorption and desorption were performed at a constant temperature.

Diffuse reflectance infrared Fourier transform spectroscopy (DRIFTS) analyses were carried out by switching the inlet flow from inert Ar stream to the adsorbing CO_2_ stream according to a published procedure [44,45]. Fourier transform infrared (FT-IR) spectra (Model spectrum TM100, Perkin Elmer Japan Co., Ltd., Tokyo, Japan) were recorded at 3 and 10 min and the maximum adsorptions were recorded after 10 min under CO_2_ atmosphere. Upon saturation of the sorbent, the inlet stream was switched back to the inert Ar stream and FT-IR desorption spectra were recorded at the same time intervals that the adsorption step. Dry CO_2_ was used for the runs and high purity Ar as the purging gas for sorbent regeneration. 

## 3. Results and Discussions

### 3.1. ROSZ Preceramic Polymers 

**Figure 2:**
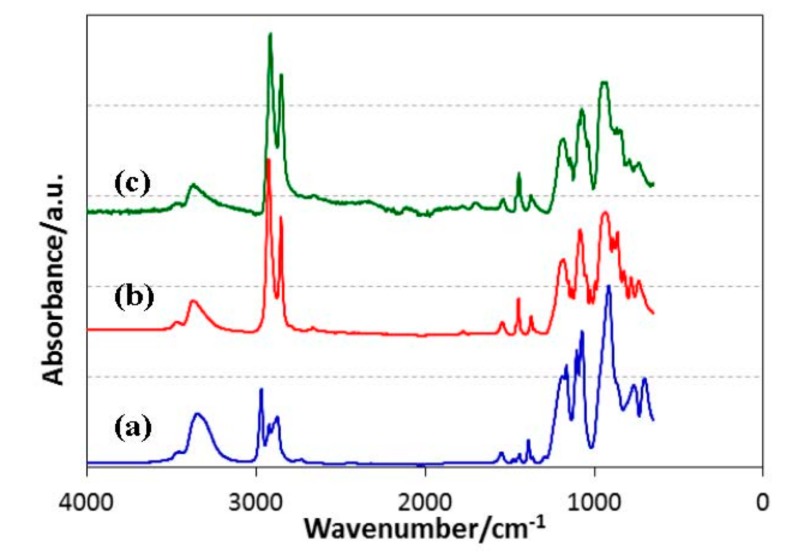
ATR-IR spectra of (a) EtOSZ; (b) CyOSZ and (c) DHNpOSZ.

Typical ATR-IR spectra of the ROSZ are shown in Figure 2. All the sample polymers exhibited characteristic absorption bands at 3350 (broad), 2800–3000 and 1070 cm^−1^ attributed to νN–H, νC–H and δN–H that were involved in Si–NH–Si unit [46], respectively. 

**Figure 3:**
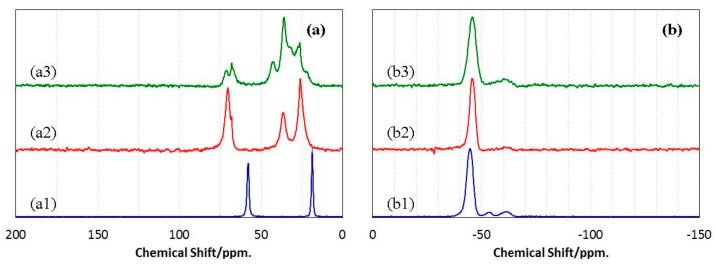
(**a**) ^13^C– and (**b**) ^29^Si–NMR spectra of as-synthesized ROSZs. (a1,b1) EtOSZ, (a2,b2) CyOSZ, and (a3,b3) DHNpOSZ.

To identify the chemical structure of the ROSZ in more detail, ^13^C– and ^29^Si–NMR spectroscopic analyses were performed in solid state. Typical results are shown in Figure 3. The ^13^C–NMR spectrum of EtOSZ (a1) presented two sharp signals at 58.0 and 18.8 ppm, assigned to methylene (CH_2_) unit, and terminate methyl (CH_3_) unit in the ethoxy (OCH_2_CH_3_) group, respectively. The corresponding ^29^Si–NMR spectrum (b1) exhibited a strong single signal at −44.6 ppm that was assigned to SiO(NH)_3_ unit. The weak signals at −53.6 and −61.4 ppm were thought to be attributed to the small amount of by-products (below 5%) that could not be removed by the distillation after the alkoxylation of SiCl_4_ (Equation (1)). The signals at −53.6 and −61.4 ppm were assigned to (EtO)_2_–Si–(NH)_2_ (linear or cyclic) and (EtO)_3_–Si–NH, respectively [24]. 

The ^13^C–NMR spectrum of as-synthesized CyOSZ (a2) showed three sharp signals at 26.2, 36.7 and 70.4 ppm assigned to the carbon atoms at the metha- and para-position (26.2 ppm), ortho-position (36.7 ppm) of the six-membered ring, and those that bonded to –OSi–NH (70.4 ppm), respectively. In the case of DHNpOSZ (a3), three sharp signals at 26.5, 36.2 and 43.0 ppm were observed together with several broad signals at 19.6 to 48.4 ppm attributed to the carbons of the bicyclo[4.4.0]decane moiety except for those that bonded to –OSi–NH observed at 68.1 and 71.3 ppm. This complicated spectrum was assumed to be due to the co-existence of the cis and trans isomers in the starting decahydro-2-naphtol used in this study. On the other hand, the corresponding ^29^Si–NMR spectra exhibited almost same feature with a strong single signal at −44.6 ppm assigned to SiO(NH)_3_ unit (b2 and b3). 

### 3.2. Thermal Conversion to Inorganic Compound

**Figure 4:**
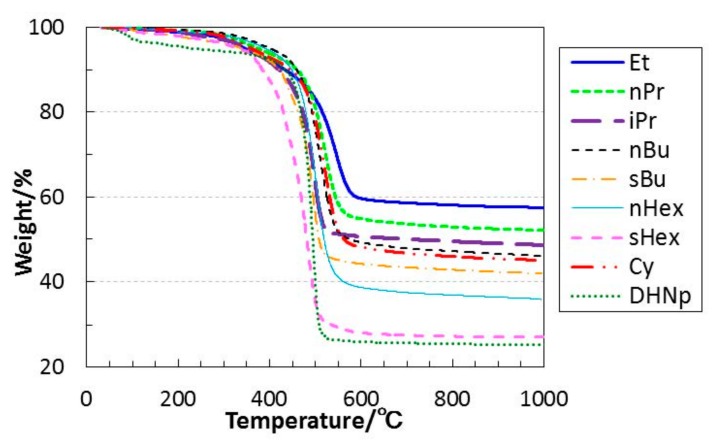
Thermogravimetric (TG) curves for as-synthesized ROSZs measured in N_2_.

As shown in Figure 4, ROSZ samples showed a similar TG curve with the following two weight loss regions: a slight weight loss of approximately 4.5% up to 200 °C, which could be due to the residual solvent, and a main weight loss at around 300 to 600 °C. The main weight loss increased consistently with the molecular weight of the alkyl group (R) of the OR group in ROSZ, and the ceramic yield evaluated for the 800 °C-pyrolyzed samples followed this relationship (Table 2). 

**Figure 5:**
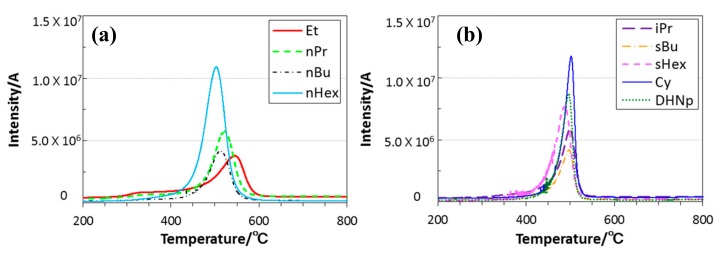
Relationship between heating temperature and total ion current chromatogram (TICC) detected for the gaseous species formed in-situ. ROSZ sample polymers having (**a**) primary alkyl group; and (**b**) secondary alkyl group.

**Figure 6:**
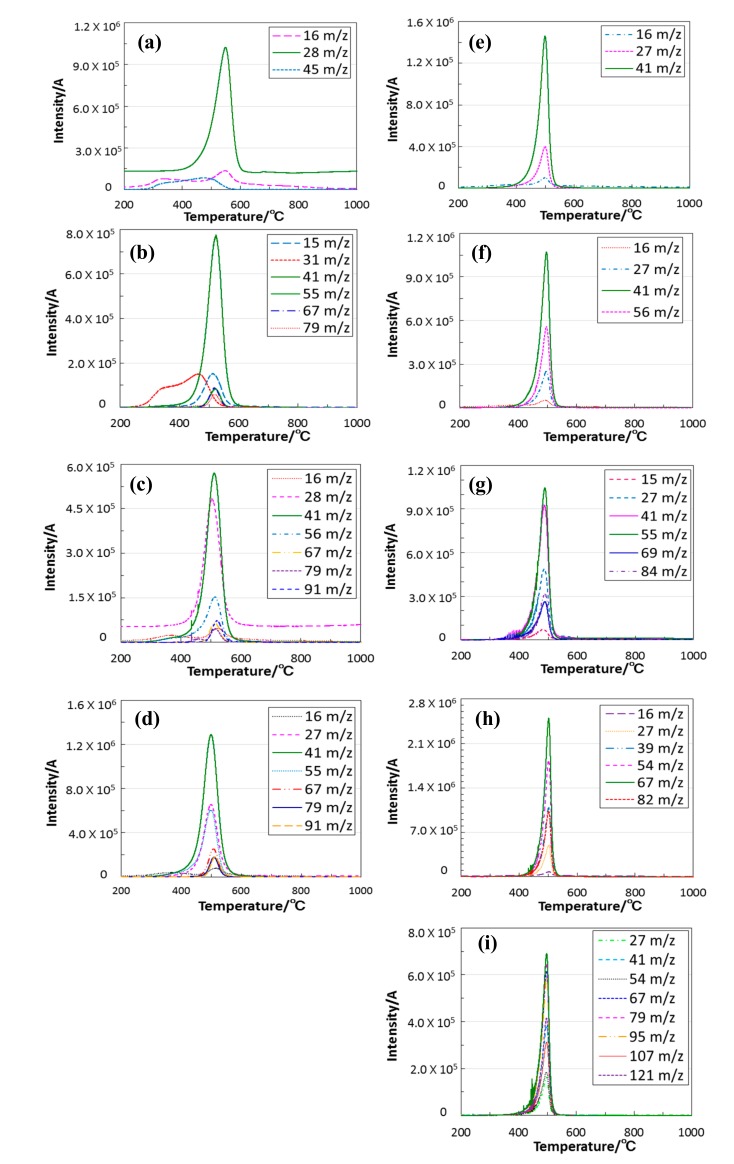
Continuous in-situ monitoring of the evolved gaseous species by mass spectroscopy. ROSZ sample polymers with R = (**a**) Et [22]; (**b**) nPr; (**c**) nBu; (**d**) nHex; (**e**) iPr; (**f**) sBu; (**g**) sHex; (**h**) Cy and (**i**) DHNp.

Chemical compositions of the 800 °C-pyrolyzed ROSZ samples were listed in Table 3. As a reference data, the theoretical compositions of the as-synthesized ROSZ polymers were also listed in this table. In spite of the pyrolysis under inert atmosphere of N_2_, the carbon content remarkably decreased, and the resulting C/Si atomic ratios of the 800 °C-pyrolyzed ROSZ samples were in the range of 0 to 0.08. The nitrogen content also decreased to some extent, and the resulting N/Si atomic ratios were 0.5 to 0.6, while the O/Si atomic ratios were 0.8 to 1.1, close to that of the ideal ROSZ (1.0). Then, TG-MS analysis was performed on the as-synthesized ROSZ samples under He atmosphere. The results were summarized and shown in Figure 5 and Figure 6. 

The TG-curves measured in He were quite similar to those in N_2_ (Figure 4), and the gaseous species formed in-situ were mainly detected during the dominant weight loss region at 300 to 600 °C, however, compared with the ROSZs having primary alkyl group (Figure 5a), those with secondary alkyl group showed a narrow TICC signal and the gas evolution completed up to 550 °C (Figure 5b).

As previously reported [24], the gaseous species, *m*/*z* ratios at 45 and 16 detected for EtOSZ (Figure 6a) were assigned to SiNH_3_^+^ and NH_2_^+^, respectively. These fragment ions could be due to the partial decomposition of the silsesquiazane linkage. This led to the lower nitrogen content observed for the 800 °C-pyrolysed EtOSZ. The *m*/*z* ratio at 28 was assigned to ethylene (CH_2_ = CH_2_^+^) formed in-situ by the C–O bond cleavage of the ethoxy group.

As shown in Figure 6, the *m*/*z* ratios at 16 (NH_2_^+^) and/or 15 (NH^+^) were detected for other ROSZ analogues, and the partial decomposition of the silsesquiazane linkage could proceed to some extent in all the ROSZ samples synthesized in this study. 

Together with these species, gaseous species detected at various *m*/*z* ratios are listed in Table 4. As we expected, other *m*/*z* ratios detected for the ROSZ analogues with secondary alkyl group (R = iPr, sBu, sHex, Cy and DHNp) were basically assigned as the fragment ions of the corresponding alkenes (C(R^1^) = CH–R^2+^) [47] shown in Equation (1). 

–Si–O–CH(R^1^)–CH_2_–R^2^ → –SiO^●+^ + C(R^1^) = CH–R^2 +^ + 1/2H_2_ (1)

R^1^, R^2^ = H, alkyl

In the case of ROSZ analogues with primary alkyl group (R = nPr, nBu, nHex), this C–O bond cleavage could proceed in association with some Si–N bond cleavages. The unknown species such as *m*/*z* ratios at 67, 79 and 91 were estimated as oligomers originated from the silsesquiazane linkage. 

### 3.3. Textural Properties of ROSZ-Derived Amorphous Si–O–N

To study the porous structure formation, textural properties of the ROSZ-derived amorphous Si–O–N samples were characterized by measuring gas adsorption-desorption isotherm. Based on the results of TG and TG-MS analyses, the following three kinds of ROSZ analogues with R = Et, Cy and DHNp were selected. Then, the porosity formation behavior was studied by evaluating the Brunauer–Emmet–Teller (BET) specific surface area (SSA) of the ROSZ-derived Si–O–N samples pyrolyzed at various temperatures from 400 to 800 °C. 

**Figure 7:**
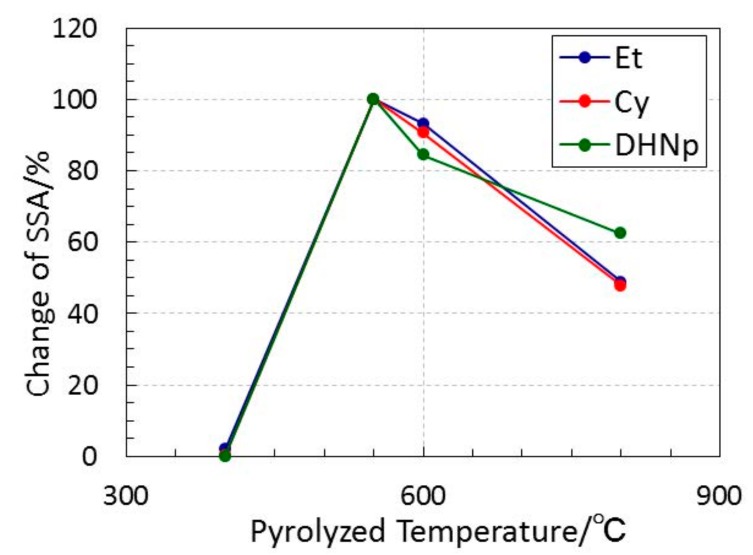
Relative specific surface areas (SSAs) evaluated for ROSZ-derived amorphous Si–O–N samples as a function of pyrolysis temperature.

XRD analysis of the 800 °C-pyrolyzed samples resulted in the detection of a typical amorphous diffraction line, and as shown Figure 7, the SSA of all the X-ray amorphous samples drastically increased above 400 °C to reach its maximum at 550 °C, then decreased consistently with the pyrolysis temperature. The immediate increase in the SSA at 400 to 550 °C was well consistent with the gas evolution behavior of ROSZs. Then, the resulting porous structure was examined for each sample just after the pyrolysis at the gas evolution complete temperature identified and shown in Figure 5, 600 °C for EtOSZ and 550 °C for CyOSZ and DHNpOSZ. 

**Figure 8:**
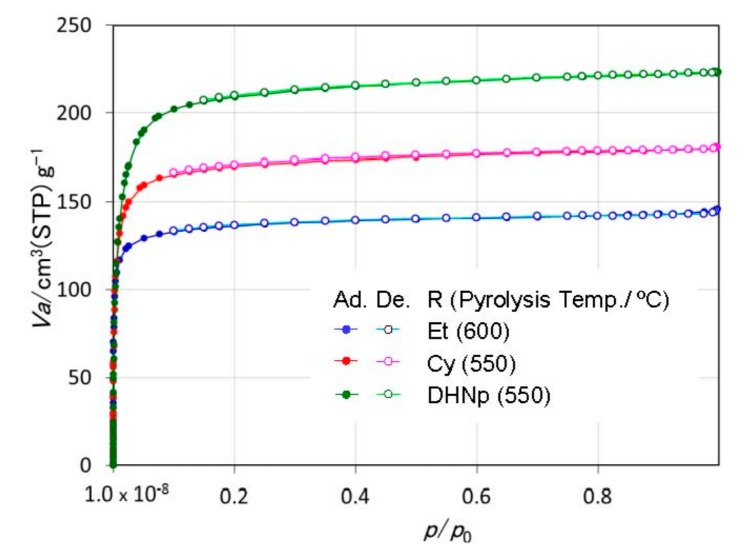
Ar sorption isotherms of ROSZ-derived amorphous Si–O–N samples.

**Figure 9:**
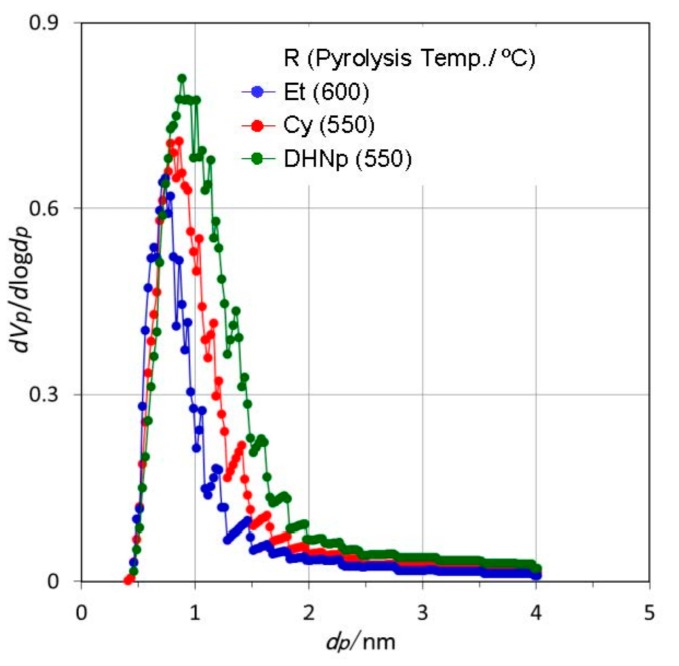
Micropore size distribution of ROSZ-derived amorphous Si–O–N samples.

The SSAs of the 600 °C-pyrolyzed EtOSZ, 550 °C-pyrolyzed CyOSZ and DHNpOSZ were measured to be 476, 601 and 750 m^2^·g^−1^, respectively (Table 5). These three samples exhibited a typical type I isotherm (Figure 8) and a unimodal pore size distribution (PSD) curve (Figure 9). The peak top of the PSD curve located in the micropore size range, and the micropore volume evaluated for these samples were listed in Table 5.

**Figure 10:**
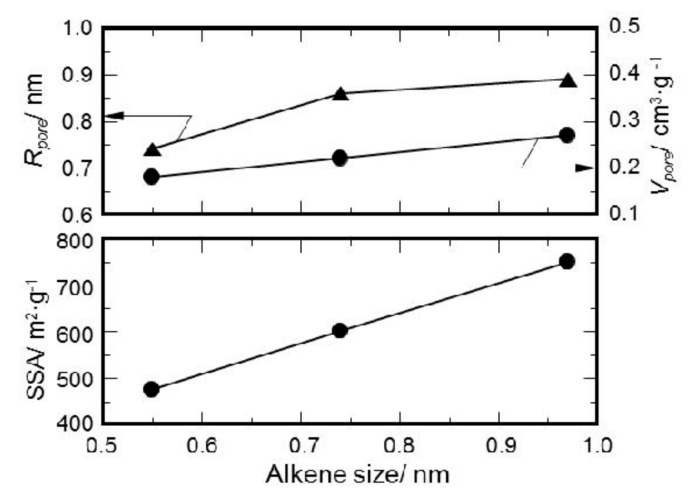
Textural properties of ROSZ-derived amorphous Si–O–N samples as a function of an estimated molecular size of alkene formed in-situ during pyrolysis in N_2_.

To study the effect of the molecular size of gaseous species formed in-situ on the porosity formation, molecular structure calculations were performed on the alkenes detected as a main gaseous component. The sizes obtained for ethylene, cyclohexene and octahydronaphthalene were 0.55, 0.74 and 0.97 nm, respectively. Then, the values listed in Table 5 were plotted as a function of the estimated molecular size of alkene (Figure 10). It was clarified that the SSA and micropore volume linearly increased with the molecular size of alkene. The peak top of the PSD curve also exhibited a similar correlation with the molecular size. 

### 3.4. CO_2_ Capture Properties 

Generally, CO_2_ capture capacity strongly depends on the SSA of solid sorbent [15,44,45] and, in this study, evaluation was performed on the three samples; 550 °C-pyrolyzed DHNpOSZ having the highest SSA together with 600 °C- and 800 °C-pyrolyzed EtOSZ samples.

In order to determine the nature of the interactions occurring at the surface of the present ROSZ-derived amorphous Si–O–N samples, CO_2_ adsorption/desorption isotherms were carried out at 0, 20 and 40 °C. 

**Figure 11:**
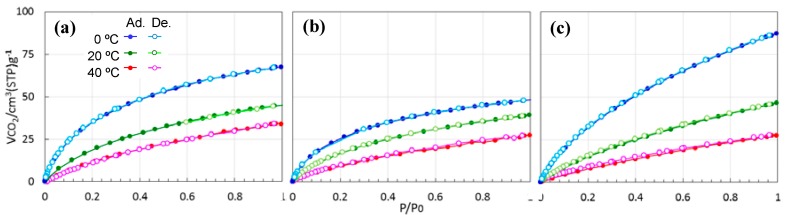
Temperature dependence of CO_2_ isotherm for (**a**) EtOSZ pyrolyzed at (**a**) 600 °C; (**b**) 800 °C and (**c**) DHNpOSZ pyrolyzed at 550 °C.

**Figure 12:**
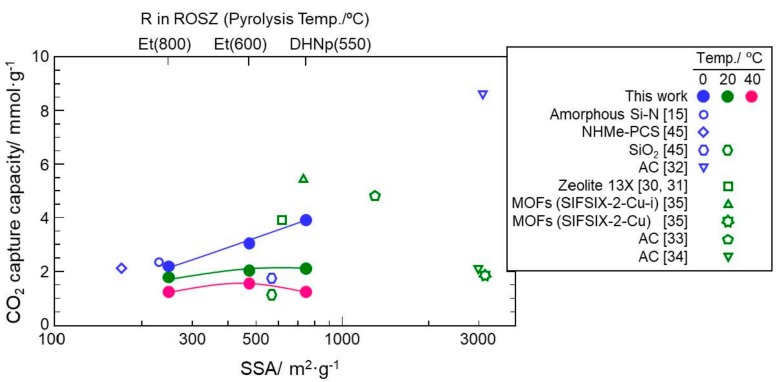
CO_2_ capture capacity vs. SSA of ROSZ-derived amorphous Si–O–N samples.

As shown in Figure 11, all the Si–O–N samples showed common features identified basically as physisorption: (1) The amount of CO_2_ up-take increased consistently with CO_2_ partial pressure, and (2) The amount of CO_2_ up-take decreased with increasing temperature. Then, the CO_2_ adsorption capacities evaluated at the CO_2_ partial pressure, *p*/*p*_0_ = 1.0 were plotted as a function of SSA of the ROSZ-derived Si–O–N samples (Figure 12). In this figure, CO_2_ adsorption capacities at 1 bar of some representative solid sorbents reported in the scientific literature [15,30,31,32,33,34,35,45] were also plotted. At 0 °C, the CO_2_ capture capacity of the 800 °C-pyrolyzed EtOSZ (Et(800)) was 2.16 mmol·g^−1^ at SSA = 279 m^2^·g^−^^1^. This value was compatible with that of polymer-derived amorphous Si–N (2.35 mmol·g^−1^ at SSA = 230 m^2^·g^−^^1^) [15] and/or methylamine-fuctionalized polycarbosilane having chemisorption property (2.1 mmol·g^−1^ at SSA = 170 m^2^·g^−^^1^) [45]. Then, ROSZ-derived Si–O–N samples linearly increased with increasing SSA, and an excellent capacity of 3.9 mmol·g^−1^ was achieved for 550 °C-pyrolyzed DHNpOSZ (DHNp(550)), although at 0 °C and 1 bar, the highest CO_2_ capture capacity of 8.64 mmol·g^−1^ was reported for activated carbons (AC) having a very large SSA of 3100 m^2^·g^−^^1^ [32]. The capacity of the DHNp(550) drastically decreased with increasing temperature. As a result, at 20 and 40 °C, the 600 °C-pyrolyzed EtOSZ (Et(600)) exhibited the highest CO_2_ capture capacity among the present three samples. 

**Figure 13:**
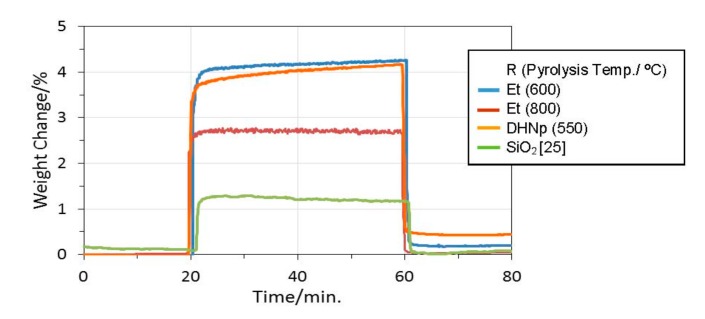
TG analysis of ROSZ-derived amorphous Si–O–N samples under continuous run Ar–CO_2_–Ar at 40 °C.

At 20 °C and 1 bar, compared with conventional Zeolite 13X (3.9 mmol·g^−1^) [30,31], MOF (SIFSIX-2–Cu–i) [35] and another AC (treated under mild condition using KOH) [33] exhibited higher capture capacities of 5.4 and 4.8 mmol·g^−1^, respectively. On the other hand, the CO_2_ capture capacity at 20 °C of the present ROSZ-derived amorphous Si–O–N was limited to be 2.0 mmol·g^−1^ at SSA = 476 m^2^·g^−^^1^ (Et(600)); however, this capacity value was found to be compatible with that of some other solid sorbents having much larger SSA, for example, AC (2.1 mmol·g^−1^ at SSA = 2994 m^2^·g^−^^1^) [34] and MOF (SIFSIX–2–Cu) (1.8 mmol·g^−1^ at SSA = 3140 m^2^·g^−^^1^) [35]. Compared with polymer-derived amorphous silica (SiO_2_) evaluated in our previous study [44,45], present EtOSZ-derived Si–O–N samples also showed higher CO_2_ adsorption capacities in spite of their lower SSAs. This seemed to be due to the capillary condensation effect derived from the microporisity of the present Si–O–N samples as previously suggested for the amorphous CN and Si–N by Zhao et al. [42] and Schitco et al. [15], respectively. However, as shown in Figure 11, such an immediate CO_2_ uptake was not clearly observed at the very low CO_2_ partial pressure region. Then, to investigate the CO_2_ capture properties of the present Si–O–N in more detail, CO_2_ adsorption/desorption behaviors were in-situ monitored by measuring TG curves under continuous run Ar–CO_2_–Ar at 40 °C. Figure 13 shows the samples weight gain under CO_2_ flow, expressed in weight percentage. A sharp weight gain was observed for all the samples. The weight gain due to the CO_2_ up-take was in the order of 600 °C-pyrolyzed EtOSZ, 550 °C-pyrolyzed DHNpOSZ and 800 °C-pyrolyzed EtOSZ, which was consistent with the result obtained by the CO_2_ sorption isotherm shown in Figure 12. In an attempt to regenerate the samples at the same temperature under Ar flow, 800 °C-pyrolyzed EtOSZ desorbed almost all its captured CO_2_, demonstrated by the total decrease in the weight gain. This desorption behavior was similar to that of SiO_2_ which had no chemical interaction towards CO_2_ [44,45]. On the other hand, the regeneration under Ar flow of the 550 °C-pyrolyzed DHNpOSZ and 600 °C-pyrolyzed EtOSZ resulted in leaving 0.43 and 0.2 wt % of the adsorbed CO_2,_ respectively, which suggesting the existence of chemical interaction toward CO_2_. 

**Figure 14:**
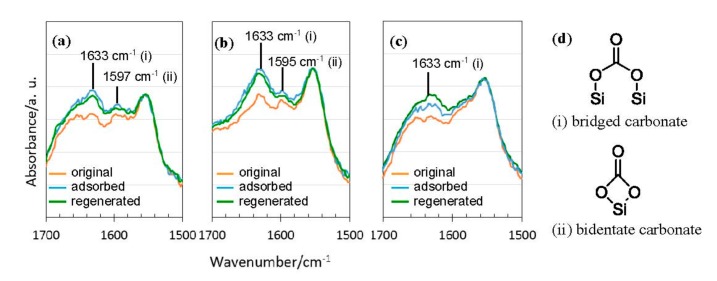
Diffuse reflectance infrared Fourier transform spectroscopy (DRIFTS) absorbance spectra before and after CO_2_ adsorption, followed by Ar regeneration on (**a**) 550 °C-pyrolyzed DHNpOSZ; (**b**) 600 °C-pyrolyzed EtOSZ; (**c**) 800 °C-pyrolyzed EtOSZ; and (**d**) proposed species formed in-situ during CO_2_ adsorption of ROSZ-derived amorphous Si–O–N samples.

The nature of the interaction was further investigated for each sample using diffuse reflectance infrared Fourier transform spectroscopy (DRIFTS). Figure 14a–c show the IR spectra of the three samples before and after CO_2_ adsorption, then after a regeneration step under Ar flow. In this study, the maximum adsorption rates were recorded after 10 min of CO_2_ exposure for the three samples. Flowing CO_2_ over 550 °C-pyrolyzed DHNpOSZ sample (Figure 14a) produced an increase in characteristic IR bands associated with the formation of proposed species shown in Figure 14d [48]. The increased intensities in the broad absorption bands at 1633 and 1597 cm^−1^ were assigned to bridged carbonate and bidentate carbonate (shown in Figure 14d), respectively. Flowing CO_2_ on 600 °C-pyrolyzed EtOSZ for 10 min also resulted in the appearance of the increased intensities in the two broad absorption bands at 1633 and 1595 cm^−1^ assigned to bridged carbonate and bidentate carbonate, respectively (Figure 14b). These samples kept the increased intensities after regeneration under Ar flow for 10 min. This was consistent with the TG analysis which showed a remaining amount of CO_2_ that could not be removed at ambient conditions. In the case of 800 °C-pyrolyzed EtOSZ, subsequent regeneration under Ar flow resulted in the further increase in the broad absorption band intensity at 1633 cm^−1^ assigned to bridged carbonate (Figure 14c).

**Figure 15:**
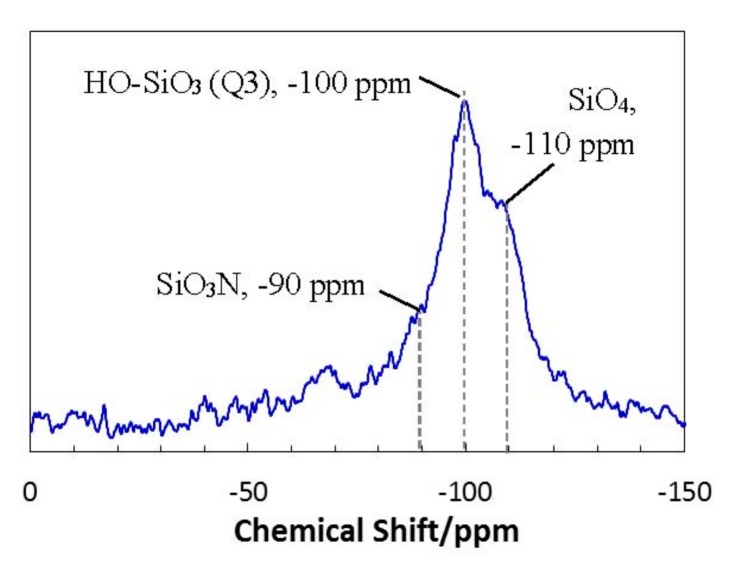
^29^Si–NMR spectrum of 800 °C-pyrolyzed EtOSZ [24].

Figure 15 shows the ^29^Si-NMR spectrum of 800 °C-pyrolyzed EtOSZ measured in our previous study [24]. One broad peak at around −100 ppm was deconvoluted to three peaks centered at −110, −100, and −90 ppm that were assigned to SiO_4_, HO–SiO_3_ (Q3), and SiO_3_N, respectively. Accordingly, the ROSZ-derived Si–O–N samples in this study could have surface OH groups.

At very low CO_2_ partial pressures, the surface OH groups provided CO_2_ chemisorption sites forming carbonates shown in Figure 14d, which could lead to enhancing CO_2_ physisorption at higher partial pressures as previously discussed for the enhanced CO_2_ capture capacity of the polycarbosilane-derived amine-functionalized Si–C–H materials [45]. The contribution of the CO_2_ chemisorption—i.e., the number of OH groups/surface area of the 550 °C-pyrolyzed DHNpOSZ—was thought to be smaller than those of two other samples. This resulted in the very high CO_2_ capture capacity at 0 °C, while the temperature dependence became more pronounced, and the CO_2_ capture capacity drastically decreased at 20 to 40 °C. 

## 4. Summary

In this study, novel polyalkoxysilsesquiazanes were synthesized. The microporosity formation behavior during the polymer/ceramics thermal conversion and the CO_2_ capture properties of the resulting X-ray amorphous microporous Si–O–N materials were investigated. The results can be summarized as follows:

(1) ATR–IR, ^13^C– and ^29^Si–NMR spectroscopic analyses revealed that a series of polyalkoxysilsesquiazanes ([ROSi(NH)_1.5_]_n_, ROSZ, R = Et, nPr, iPr, nBu, sBu, nHex, sHex, cHex, DHNp) were successfully synthesized in a good yield via two simple steps: reaction of SiCl_4_ with ROH to afford ROSiCl_3_, followed by ammonolysis at −78 °C.
(2) The simultaneous TG-MS analyses of the ROSZs under a He flow revealed that the cleavage of the oxygen–carbon bond of the RO group was a common and dominant decomposition reaction. The subsequent evolution of alkene as a main gaseous species formed in-situ lead to the formation at 550 to 800 °C of the X-ray amorphous microporous Si–O–N.
(3) The peak of the pore size distribution curve located within the micropore size range, and the total micropore volume as well as the SSA of the resulting X-ray amorphous Si–O–N increased consistently with the molecular size estimated for the alkene formed in-situ during the pyrolysis.
(4) The CO_2_ capture capacity at 0 °C of the Si–O–N material increased consistently with the SSA, and reached 3.9 mmol·g^−1^ at the SSA of 750 m^2^·g^−1^, which was achieved for the 550 °C-pyrolyzed DHNpOSZ.
(5) DRIFTS monitoring of the CO_2_ adsorption under continuous Ar–CO_2_–Ar run at 40 °C revealed carbonate formations at the CO_2_ chemisorption site provided by the surface OH groups formed in-situ during the polymer/ceramics thermal conversion at around 550 °C.
(6) The CO_2_ capture capacity measured at the CO_2_ partial pressure, *p/p*_0_ = 1 could be enhanced by the initial CO_2_ chemisorption at very low CO_2_ partial pressures.

## Figures and Tables

**Table 1:** ROSZs synthesized in this study. DHNp: decahydronaphthyl.

R	ROSiCl_3_		[ROSi(NH)_1.5_]_n_		Total Yield/%
	b.p.	Recovery Rate/%	Appearance	Yield/%	
Et	102 °C/760 mmHg	60	Colorless solid	95	57
nPr	123 °C/760 mmHg	51	Colorless solid	87	44
iPr	116 °C/760 mmHg	61	Colorless solid	93	57
nBu	150 °C/760 mmHg	53	Colorless solid	94	50
sBu	175 °C/760 mmHg	64	Colorless paste	73	47
nHex	135 °C/32 mmHg	57	Colorless paste	86	49
sHex	120 °C/24 mmHg	54	Colorless paste	88	48
Cy	78 °C/11 mmHg	53	Colorless solid	98	52
DHNp	185 °C/16.5 mmHg	52	Colorless solid	97	51

**Table 2:** Ceramic yield and appearance of 800 °C-pyrolyzed ROSZ.

R	Yield/%	Appearance
Et	58	Brown solid
nPr	53	Black solid
iPr	50	Colorless solid
nBu	47	Black solid
sBu	43	brown solid
nHex	37	Black solid
sHex	28	Brown solid
Cy	46	Pale brown solid
DHNp	26	Black solid

**Table 3:** Chemical composition of 800 °C-pyrolyzed ROSZ samples.

R	Treated Temperature	Composition/wt %					Empirical Ratio
		Si	C	O	N	H	
Et	As-synthesized	29.3	25.1	16.8	22.0	6.8	Si_1.0_C_2.0_O_1.0_N_1.5_H_6.5_
	800 °C-pyrolysed	51.3	1.1	32.4	13.5	1.7	Si_1.0_C_0.05_O_1.1_N_0.5_H_0.9_
iPr	As-synthesized	25.6	32.9	14.6	19.2	7.8	Si_1.0_C_3.0_O_1.0_N_1.5_H_8.5_
	800 °C-pyrolysed	52.2	0.04	32.4	14.0	1.4	Si_1.0_C_0.0_O_1.1_N_0.5_H_0.8_
nBu	As-synthesized	22.7	38.9	13.0	17.0	8.5	Si_1.0_C_4.0_O_1.0_N_1.5_H_10.5_
	800 °C-pyrolysed	55.1	2.6	27.2	13.7	1.4	Si_1.0_C_0.1_O_0.9_N_0.5_H_0.7_
sBu	As-synthesized	22.7	38.9	13.0	17.0	8.5	Si_1.0_C_4.0_O_1.0_N_1.5_H_10.5_
	800 °C-pyrolysed	55.0	0.2	29.3	14.2	1.4	Si_1.0_C_0.01_O_0.9_N_0.5_H_0.7_
nHex	As-synthesized	18.5	47.5	10.6	13.9	9.5	Si_1.0_C_6.0_O_1.0_N_1.5_H_14.5_
	800 °C-pyrolysed	55.2	2.7	26.4	14.6	1.23	Si_1.0_C_0.1_O_0.8_N_0.5_H_0.6_
sHex	As-synthesized	18.5	47.5	10.6	13.9	9.5	Si_1.0_C_6.0_O_1.0_N_1.5_H_14.5_
	800 °C-pyrolysed	52.3	0.8	28.2	17.0	1.3	Si_1.0_C_0.03_O_0.9_N_0.6_H_0.7_
Cy	As-synthesized	18.7	48.2	10.7	14.0	8.4	Si_1.0_C_6.0_O_1.0_N_1.5_H_12.5_
	800 °C-pyrolysed	49.9	0.10	32.9	15.7	1.4	Si_1.0_C_0.03_O_0.9_N_0.6_H_0.8_
DHNp	As-synthesized	13.8	59.0	7.9	10.3	9.1	Si_1.0_C_10.0_O_1.0_N_1.5_H_18.5_
	800 °C-pyrolysed	51.1	1.92	33.4	12.1	1.4	Si_1.0_C_0.08_O_1.1_N_0.5_H_0.8_

**Table 4:** Results of in-situ TG-MS analysis during pyrolysis up to 1000 °C under a He flow.

R	Completed Temp/°C	*m*/*z*^+^ Ratios (Assignment)
Et [24]	600	16 (NH_2_^+^), 45 (SiNH_3_^+^) 28 (ethylene)
nPr	600	15 (NH^+^) 41 (propylene) 31, 55, 67, 79 (unknown)
iPr	550	16 (NH_2_^+^) 27, 41 (propylene)
nBu	600	16 (NH_2_^+^) 28, 41, 56 (1-butene) 67, 79, 91 (unknown)
sBu	550	16 (NH_2_^+^) 27, 41, 56 (2-butene)
nHex	600	16 (NH_2_^+^) 27, 41, 55 (1- or 2-hexene) 67, 79, 91 (unknown)
sHex	550	15 (NH^+^) 27, 41, 55, 69, 84 (3-hexene)
Cy	550	16 (NH_2_^+^) 27, 39, 54, 67, 82 (cyclohexene)
DHNp	550	27, 41, 67, 79, 95, 121, 136 (1,2,3,4,4a,5,6,8a-octahydronaphthalene isomers) 54, 107 (unknown)

**Table 5:** Textural properties of ROSZ-derived amorphous Si–O–N samples.

R	Pyrolyzed Temp/°C	SSA/m^2^·g^−1^	*R_pore_*/nm	*V_pore_*/cm^3^·g^−1^
Et	600	476	0.74	0.18
Cy	550	601	0.86	0.22
DHNp	550	750	0.89	0.27
